# Leptospirosis in Urban Wild Boars, Berlin, Germany

**DOI:** 10.3201/eid1305.061302

**Published:** 2007-05

**Authors:** Andreas Jansen, Enno Luge, Beatriz Guerra, Petra Wittschen, Achim D. Gruber, Christoph Loddenkemper, Thomas Schneider, Michael Lierz, Derk Ehlert, Bernd Appel, Klaus Stark, Karsten Nöckler

**Affiliations:** * Robert Koch Institute, Berlin, Germany; † Federal Institute for Risk Assessment, Berlin, Germany; ‡ Freie Universität Berlin, Berlin, Germany; § Charité, Campus Benjamin Franklin, Berlin, Germany; ¶ Senate Department of Urban Development, Berlin, Germany

**Keywords:** Leptospirosis, Sus scrofa, urban, wildlife, zoonoses, dispatch

## Abstract

We found antibodies to leptospires in 25 (18%) of 141 wild boars from Berlin (95% confidence interval 12–25). Seropositivity was associated with chronic interstitial nephritis (odds ratio 10.5; p = 0.01), and leptospires were detected in kidney tissues. Wild boars represent a potential source for human leptospirosis in urban environments.

*Leptospira* spp. are endemic to many domestic and wild animals, which may shed the pathogen in urine (*1*). Humans may acquire potentially fatal leptospirosis through contact with urine-contaminated water or soil. In Germany, ≈50 cases of leptospirosis are reported each year, mostly related to recreational and residential exposure (*2*). Among wildlife species, rodents are considered to be the most important reservoirs for leptospirosis in rural and urban environments (*3*,*4*). Contact with water contaminated with rodents’ urine (usually inadequately treated sewage) is a well-known risk factor for leptospirosis. In wild boars (*Sus scrofa*) in Europe and the United States, antibodies against *Leptospira* spp. serovar Pomona—the main serogroup that infects domestic swine—have been frequently detected (*5*,*6*). During the past decades, the population density of this game species has increased substantially (*7*,*8*). Subsequently, boar migration to urban areas and close contact with humans has been noted. At present, an estimated 5,000 wild boars live in urban and suburban areas of Berlin. Although boars are known to be susceptible to leptospirosis, data on the prevalence of the disease in synanthropic wild boars and the possible implications for human health are absent. Our objective was to assess the potential role of wild boars as a reservoir for leptospirosis in an urban environment. In addition, we examined their role in transmission of *Leptospira* spp. to city residents with occupational exposure to wild boars.

## The Study

The survey was conducted in Berlin, which has an area of 891.7 km^2^ and a population of 3.4 million. The study area is mostly urban (56%) and contains industrial, commercial, and residential buildings; the other portions are forest (17.9%), green space (14.5%), and water (6.6%). The southwestern study area partially merges with the adjacent city of Potsdam.

Serum and kidney tissue samples were collected from wild boars killed in the study area for population control during fall and winter 2005-06. Wild boars were categorized according to age (determined by teeth; shoats <1 year, yearlings 1–2 years, adults >2 years), sex, and location of death. For antibody detection, microscopic agglutination test was conducted with a panel of 12 leptospire serovars; a titer ≥100 was considered positive (Table 1). For histologic investigation, the tissues were fixed in 10% formalin; embedded in paraffin; and stained with hematoxylin and eosin, Masson trichrome, or Warthin-Starry silver according to standard protocols. Chronic interstitial nephritis and resultant renal fibrosis, the characteristic lesions of renal leptospirosis in animals (*9*), were the criteria used to classify renal infection. Selected tissue samples were further studied by classic PCR targeting the leptospiral outer membrane lipoprotein LipL32 (*10*). The amplification products were confirmed by direct sequencing.

**Table 1:** Characteristics and titers of antibodies to leptospires of 25 seropositive wild boars, Berlin, fall/winter, 2005–06

Boar characteristics			*Leptospira* spp. serovar*						
Sex†	Age, y‡	Place of death§	Australis	Autumnalis	Bratislava	Copenhageni	Grippotyphosa	Pomona	Pyrogenes
M	Adult	1	–	–	100	–	–	–	100
M	Adult	1	–	–	100	–	–	–	–
F	Adult	1	–	–	100	–	–	–	100
M	Adult	1	–	100	800	200	200	800	–
M	Yearling	1	–	–	200	–	100	100	–
M	Adult	1	–	–	200	–	–	200	–
M	Yearling	1	–	–	200	–	100	400	–
F	Yearling	1	–	–	100	–	–	100	–
M	Shoat	1	–	–	–	–	–	400	–
M	Adult	2	–	–	–	–	–	100	–
F	Shoat	2	–	–	–	–	–	100	–
F	Yearling	3	–	–	100	–	–	–	–
F	Adult	3	–	–	–	–	–	200	–
F	Yearling	3	–	–	–	–	100	–	–
F	Yearling	3	–	–	100	–	100	400	–
F	Adult	3	–	–	–	–	–	–	100
F	Adult	3	100	–	100	–	–	–	100
M	Yearling	3	–	–	–	–	–	400	–
NK	Yearling	4	–	–	100	–	–	–	–
F	Shoat	4	–	–	–	–	–	–	100
F	Adult	5	–	–	100	–	–	–	–
M	Shoat	6	–	–	100	–	–	–	100
F	Shoat	7	400	–	–	–	–	–	–
F	Shoat	7	–	–	–	–	–	800	–
M	Adult	7	–	–	–-	–-	–	–	100

**Figure 1:**
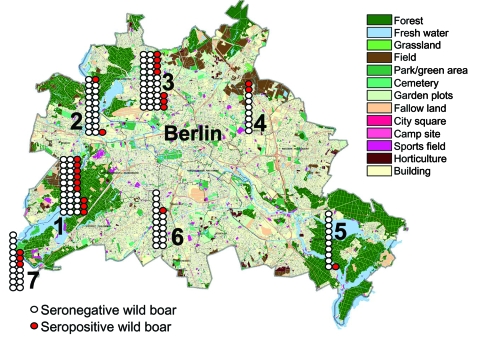
Map of Berlin showing the regional distribution, numbers of wild boars killed during the 2005–06 hunting season (n = 141), and numbers of wild boars seropositive for antibodies against *Leptospira* spp. (red). Districts are numbered from 1 to 7 (with permission from the Senate Department of Urban Development, Berlin) and correspond to numbers on Tables 1 and 2.

*Microscopic agglutination test panel included *Leptospira interrogans* serovars Australis, Autumnalis, Bataviae, Bratislava, Copenhageni, Hardjo, Icterohaemorrhagiae, Pomona, and Pyrogenes; *Leptospira borgpetersenii* serovar Tarassovi; and *Leptospira kirschneri* serovar Grippotyphosa. A titer of ≥100 or above was considered positive. The highest titers are shown.  †NK, not known. ‡Shoat, <1 y; yearling, 1–2 y; adult >2 y. §Numbers correspond to areas shown in Figure 1.

To assess seroprevalence among city residents, in April 2006 we enrolled 84 municipal hunters from Berlin. A standardized questionnaire was used to assess demographic information (age, sex), frequencies of contact with wild boars, and use of gloves during boar evisceration. Serum samples were collected from all hunters and analyzed as described above. Univariate odds ratios (OR), prevalence ratios (PR), and 95% confidence intervals (95% CI) were calculated by using SPSS 14 software (SPSS Inc., Chicago, USA). A p value <0.05 was considered significant.

**Figure 2:**
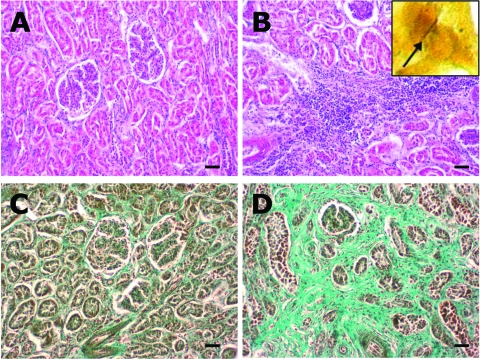
A) Normal renal parenchyma from wild boar seropositive for *Leptospira* spp. (hematoxylin and eosin [HE] staining). B) Kidney from a seropositive wild boar, showing chronic interstitial nephritis (HE staining). Inset: silver-stained leptospire (arrow) within the tubulus epithelium of the kidney (Warthin-Starry, oil ×1,000). C) Normal renal parenchyma (Masson trichrome staining). D) Kidney with severe interstitial fibrosis (green) as a result of chronic interstitial nephritis in a wild boar seropositive for *Leptospira* spp. (Masson trichrome staining). Scale bars represent 50 μm.

During the 2005–06 hunting season, municipal hunters in the study area shot 294 wild boars. A total of 219 (74%) blood samples and 77 (26%) kidney specimens were collected from the boars. Of these, 78 (36%) blood samples had to be excluded from analysis because of insufficient quality (i.e., clotting). Of the 141 remaining serum samples, antibodies against pathogenic leptospires were found in 25 (18%) (95% CI 12–25). Of these 25 positive samples, 10 demonstrated cross-reactivity with antigens of other serovars. Among the samples without cross-reactivity, *Leptospira* spp. serovar Pomona (n = 6) and serovar Bratislava (n = 4) were most frequently identified (Table 1). Titers of *Leptospira-*positive serum samples varied from 100 through 800. Although not statistically significant, seropositivity was highest in animals from the southwestern part of the study area (Figure 1; Table 2). Seropositivity was also higher in adult animals (p = 0.02) but was unrelated to sex (p = 0.6). A total of 29 kidney specimens were examined histologically, 17 of which were from seropositive boars. Of these17, 15 (88%) showed moderate to severe chronic lymphoplasmacytic interstitial inflammation (Figure 2), compared with 5 (42%) from the 12 seronegative boars (OR 10.5; 95% CI 1.3–110.4; p = 0.01). Leptospires were detected by silver staining in 3 (30%) of 10 specimens from seropositive wild boars with chronic interstitial nephritis (5 of the 15 specimens were unsuitable for silver staining) and were confirmed by PCR in 2 of the leptospire-positive samples. However, among 84 municipal hunters (96% males, mean age 51 years) antibodies to leptospires were not detected. Sixty-one (73%) hunters shot >10 wild boars per season. Of these, 51 (72%) used gloves, at least on occasion, compared with 6 of the 17 hunters (35%) who shot ≤10 boars per year (PR 2; 95% CI 1.2–3.4; p<0.05).

**Table 2:** Locations of wild boars seropositive for *Leptospira* spp., Berlin, fall/winter, 2005–06*

District†	% Boars	95%CI
1. Zehlendorf/Wannsee	27	15–44
2. Spandau	10	3–29
3. Tegel/Reinickendorf	21	11–38
4. Pankow/Mahrzahn/Hellersdorf	22	6–55
5. Köpenick	9	2–38
6. Lichterfelde/Steglitz/Charlottenburg	16	1–27
7. Potsdam	18	6–41
Total	18	12–25

*Seropositivity determined by microscopic agglutination test. CI, confidence interval.  †Numbers correspond to areas shown on Figure 1.

## Conclusions

Our study describes a newly discovered urban focus of leptospirosis among wild boars in Berlin. The high frequency of porcine *Leptospira* spp. serovars Pomona and Bratislava, the association of chronic interstitial nephritis with positive results of the microscopic agglutination test, and the demonstration of leptospires in kidney specimens all indicate that wild boars act as a maintenance host for *Leptospira* spp. in this urban area. A relatively high prevalence of leptospirosis was observed in the southwestern districts of the city, which are particularly rich in freshwater lakes intensively used for recreational activities by urban inhabitants. The relevance of this finding to human health was demonstrated by a recent case of severe leptospirosis in this area of Berlin; the patient had had contact with fresh water, which was most likely contaminated by wild boar urine (*11*).

In contrast to findings of a study from Austria (*12*), we found no antibodies to leptospires in hunters. Although this finding may be related to the regular use of gloves by highly exposed persons, it also indicates that the transmission of leptospirosis from wild boars to humans, although present, occurs at a considerably low rate. However, the epidemic potential of infections like leptospirosis that have a basic reproduction number close to 0 (i.e., that are minimally transmissible within human populations) is largely determined by the number of introductions from the animal hosts. Thus, among other contributing factors (e.g., human population expansion and encroachment, transmission to sympatric populations of susceptible domestic animals), the ongoing increase in wild boar populations, and the shift from sylvatic to synanthropic occurrence of this game species might lead to increased leptospirosis in humans.

From a public health perspective, surveillance of leptospirosis incidence, prevalence, and serovar distribution in wild boars and humans (especially in potential high-risk groups with recreational freshwater contact) is necessary to establish the direction and the significance of this newly discovered potential exposure route. Additionally, physicians and public health authorities should be aware that bodies of fresh water in areas populated with wild boars may be contaminated with *Leptospira* spp., even if typical indicators, like rat infestations or contamination with sewage, are absent.

## References

[1] Levett PN. Leptospirosis. Clin Microbiol Rev. 2001;14:296–326. 10.1128/CMR.14.2.296-326.2001 11292640 PMC88975

[2] Jansen A, Schoneberg I, Frank C, Alpers K, Schneider T, Stark K. Leptospirosis in Germany, 1962–2003. Emerg Infect Dis. 2005;11:1048–54. 16022779 10.3201/eid1107.041172 PMC3371786

[3] Bharti AR, Nally JE, Ricaldi JN, Matthias MA, Diaz MM, Lovett MA, Leptospirosis: a zoonotic disease of global importance. Lancet Infect Dis. 2003;3:757–71. 10.1016/S1473-3099(03)00830-2 14652202

[4] Vinetz JM, Glass GE, Flexner CE, Mueller P, Kaslow DC. Sporadic urban leptospirosis. Ann Intern Med. 1996;125:794–8. 8928985 10.7326/0003-4819-125-10-199611150-00002

[5] Mason RJ, Fleming PJ, Smythe LD, Dohnt MF, Norris MA, Symonds ML. *Leptospira interrogans* antibodies in feral pigs from New South Wales. J Wildl Dis. 1998;34:738–43. 9813843 10.7589/0090-3558-34.4.738

[6] Vicente J, Leon-Vizcaino L, Gortazar C, Jose Cubero M, Gonzalez M, Martin-Atance P. Antibodies to selected viral and bacterial pathogens in European wild boars from southcentral Spain. J Wildl Dis. 2002;38:649–52. 12238391 10.7589/0090-3558-38.3.649

[7] Schley L, Roper TJ. Diet of wild boar *Sus scrofa* in Western Europe, with particular reference to consumption of agricultural crops. Mammal Rev. 2003;33:43–56 . 10.1046/j.1365-2907.2003.00010.x

[8] Witmer GW, Sanders RB, Taft AC. Feral swine—are they a disease threat to livestock in the United States? Fagerstone KA, Witmer GW, editors. Proceedings of the 10th Wildlife Damage Management Conference; 2003. pp. 316–25.

[9] Baker TF, McEwen SA, Prescott JF, Meek AH. The prevalence of leptospirosis and its association with multifocal interstitial nephritis in swine at slaughter. Can J Vet Res. 1989;53:290–4. 2766150 PMC1255713

[10] Levett PN, Morey RE, Galloway RL, Turner DE, Steigerwalt AG, Mayer LW. Detection of pathogenic leptospires by real-time quantitative PCR. J Med Microbiol. 2005;54:45–9. 10.1099/jmm.0.45860-0 15591254

[11] Jansen A, Nöckler K, Schönberg A, Luge E, Ehlert D, Schneider T. Wild boars as possible source of hemorrhagic leptospirosis in Berlin, Germany. Eur J Clin Microbiol Infect Dis. 2006;25:544–6. 10.1007/s10096-006-0174-3 16896826

[12] Deutz A, Fuchs K, Schuller W, Nowotny N, Auer H, Aspock H, Seroepidemiological studies of zoonotic infections in hunters in southeastern Austria—prevalences, risk factors, and preventive methods. Berl Munch Tierarztl Wochenschr. 2003;116:306–11. 12894685

